# From Septic Shock to Hemorrhagic Shock: A Rare Presentation of Ischemic Rectal Ulcer in a Critically Ill Patient

**DOI:** 10.7759/cureus.108915

**Published:** 2026-05-15

**Authors:** Wafaa Mansour, Ghaid Touza, Ali Soueidan

**Affiliations:** 1 Internal Medicine, Henry Ford Health System, Southfield, USA; 2 Critical Care Medicine, Henry Ford Health System, Southfield, USA

**Keywords:** acute gi bleed, critical illness, hemorrhagic shock, ischemic rectal injury, massive transfusion, medical intensive care unit (micu), rectal ulcer, septic shock, vasopressor, vasopressor therapy

## Abstract

Acute hemorrhagic rectal ulcer (AHRU) is an uncommon but clinically significant cause of lower gastrointestinal bleeding in critically ill patients. While stress-related mucosal disease is a well-recognized cause of upper gastrointestinal bleeding in the intensive care unit (ICU), ischemic injury involving the rectum is less frequently reported. Patients recovering from septic shock, particularly those requiring prolonged vasopressor therapy, remain at risk for delayed ischemic complications. We present a case of delayed severe lower gastrointestinal bleeding due to AHRU following septic shock, highlighting the risk of ischemic complications even after apparent clinical stabilization. A 54-year-old man with insulin-dependent type 2 diabetes mellitus presented with severe diabetic ketoacidosis and was admitted to the ICU. His course was complicated by methicillin-sensitive *Staphylococcus aureus* pneumonia and septic shock requiring prolonged vasopressor support. After initial clinical improvement and transfer out of the ICU, he developed acute hematochezia with a significant decline in hemoglobin. Computed tomography angiography demonstrated active rectal bleeding. Flexible sigmoidoscopy revealed multiple rectal ulcers consistent with AHRU. Although hematin and ulcerated mucosa with stigmata of recent bleeding were identified in the rectosigmoid region, the ulcers were not actively bleeding at the time of the procedure, and no endoscopic hemostatic intervention was performed; lavage was performed to improve visualization. His condition deteriorated with hemorrhagic shock requiring reintubation, vasopressor support, and massive transfusion protocol. Following multidisciplinary management, he stabilized and was ultimately discharged to a rehabilitation facility. This case is notable for the delayed onset of severe lower gastrointestinal bleeding due to AHRU following apparent recovery from septic shock, highlighting a temporal gap between hemodynamic insult and clinical manifestation. Unlike typical stress-related mucosal disease, which primarily affects the upper gastrointestinal tract, AHRU represents a less recognized form of ischemic injury involving the rectum, likely driven by prolonged splanchnic hypoperfusion and vasopressor-induced vasoconstriction. The severity of bleeding requiring massive transfusion and critical care re-escalation underscores the potential for rapid clinical deterioration. This case emphasizes the importance of maintaining a high index of suspicion for ischemic rectal injury in post-ICU patients with new-onset hematochezia, even after initial stabilization. This case highlights a rare and delayed ischemic complication of septic shock. AHRU should be considered in critically ill patients with new-onset lower gastrointestinal bleeding, particularly following prolonged vasopressor use. Current stress ulcer prophylaxis strategies may not prevent such complications, emphasizing the need for broader preventive approaches targeting splanchnic perfusion, bowel care, minimization of prolonged rectal device use, and early recognition of ischemic lower gastrointestinal injury.

## Introduction

Acute hemorrhagic rectal ulcer (AHRU) is a rare but clinically significant cause of lower gastrointestinal bleeding in critically ill patients, particularly in the intensive care unit (ICU) setting [1]. While gastrointestinal bleeding in the ICU is more commonly attributed to upper tract pathology such as stress-related mucosal disease [2,3], lower gastrointestinal sources like AHRU remain underrecognized. Unlike acid-mediated or inflammatory injury, AHRU is primarily driven by ischemic mechanisms related to systemic hypoperfusion and critical illness [4,5]. Although the rectum has a dual blood supply, this protective feature may be insufficient during severe physiologic stress [6]. Hemodynamic instability, vasopressor use, and microcirculatory dysfunction impair splanchnic blood flow, predisposing to localized mucosal ischemia [4,7]. Clinically, AHRU often presents with sudden, large-volume hematochezia and can lead to rapid deterioration. Given its rarity and nonspecific presentation, diagnosis is frequently delayed, highlighting the need for increased awareness of AHRU as a potential source of lower gastrointestinal bleeding in critically ill patients [1,6].

## Case presentation

A 54-year-old man with insulin-dependent type 2 diabetes mellitus and asthma presented with altered mental status. On arrival, he was lethargic and unable to provide history. Physical examination revealed a patient who was alert and oriented to self only (A&O ×1). He opened his eyes only to painful stimulation and was able to state his name intermittently but was otherwise confused and unable to answer questions appropriately. On painful stimuli, he demonstrated abnormal flexion posturing. The Glasgow Coma Scale was 8. Cardiovascular examination demonstrated tachycardia with a regular rhythm, and lung auscultation was clear bilaterally. The abdomen was soft and nondistended without tenderness, and there was no lower extremity edema. Vital signs showed tachycardia (heart rate 112 beats/min; normal 60-100 beats/min) and tachypnea (respiratory rate 25-28 breaths/min; normal 12-20 breaths/min), with adequate oxygen saturation on 4 L/min nasal cannula. 

Laboratory evaluation was consistent with severe diabetic ketoacidosis (DKA) (Table 1), characterized by hyperglycemia (glucose >250 mg/dL; normal 70-140 mg/dL), metabolic acidosis (arterial pH <7.30; normal 7.35-7.45), low serum bicarbonate (<18 mEq/L; normal 22-28 mEq/L), and elevated anion gap (>12 mEq/L; normal 8-12 mEq/L). There was no leukocytosis (white blood cell count within 4.0-11.0 ×10⁹/L) or anemia (hemoglobin within 13.5-17.5 g/dL). Electrocardiography revealed sinus tachycardia (Figure 1). 

**Table 1:** Initial Laboratory Findings Consistent with Severe Diabetic Ketoacidosis Laboratory values on admission demonstrating severe metabolic acidosis with elevated anion gap, hyperglycemia, ketonemia, and associated electrolyte abnormalities consistent with diabetic ketoacidosis. Corrected sodium accounts for hyperglycemia.

Parameter	Value	Reference Range
pH	7.08	7.35–7.45
pCO₂	23 mm Hg	35–45 mm Hg
Glucose	634 mg/dL	70–140 mg/dL
Sodium	157 mmol/L (corrected ~166 mmol/L)	135–145 mmol/L
Potassium	5.9 mmol/L	3.5–5.0 mmol/L
Bicarbonate	6 mmol/L	22–28 mmol/L
Anion Gap	44	8–12 mEq/L
Beta-hydroxybutyrate	15.6 mmol/L	<0.5 mmol/L
Creatinine	3 mg/dL	0.7–1.3 mg/dL

**Figure 1:**
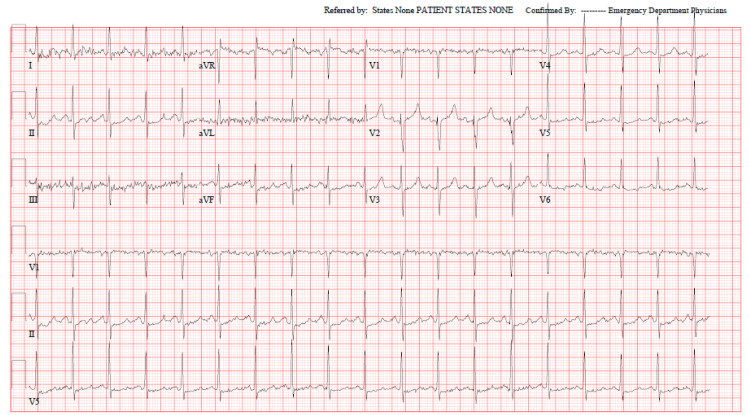
Electrocardiogram on Presentation Twelve-lead electrocardiogram demonstrating sinus tachycardia with a ventricular rate of approximately 110–120 beats per minute. P waves are present before each QRS complex with a regular rhythm. No acute ST-segment elevations or depressions are identified. No significant T-wave inversions or conduction abnormalities are noted.

He was admitted to the ICU and intubated for worsening mental status and inability to protect his airway, with aspiration occurring during intubation. During this initial ICU admission, he was started on gastrointestinal prophylaxis with pantoprazole 40 mg daily. On hospital day 2, he developed fever (maximum temperature 102.1°F; normal 97-99°F) and worsening tachycardia (heart rate in the 140s). Computed tomography of the chest demonstrated right-sided pulmonary consolidations (Figure 2).

**Figure 2:**
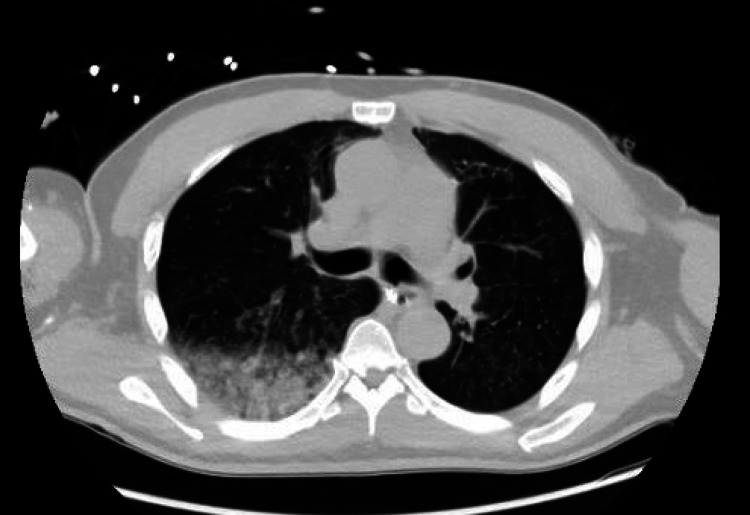
Computed Tomography of the Chest Demonstrating Right Lower Lobe Consolidation Axial contrast-enhanced computed tomography of the chest demonstrating consolidation in the dependent posterior right lung, consistent with pneumonia.

Broad-spectrum antibiotics were initiated. Blood and sputum cultures grew methicillin-sensitive *Staphylococcus aureus*, and bronchoscopy with bronchoalveolar lavage confirmed infection. Antibiotics were subsequently narrowed to cefazolin. 

His DKA resolved with continued management. However, his course was complicated by septic shock requiring vasopressor support, including norepinephrine for more than one week and vasopressin for two days. His respiratory status improved, and he was successfully extubated on hospital day 7 and transferred out of the ICU on hospital day 8. 

On hospital day 9, the patient developed acute hematochezia accompanied by worsening dyspnea. Hemoglobin decreased from 11.1 g/dL (normal 13.5-17.5 g/dL) to 7.9 g/dL. Computed tomography angiography of the pelvis demonstrated active bleeding within the mid-to-distal rectum (Figure 3).

**Figure 3:**
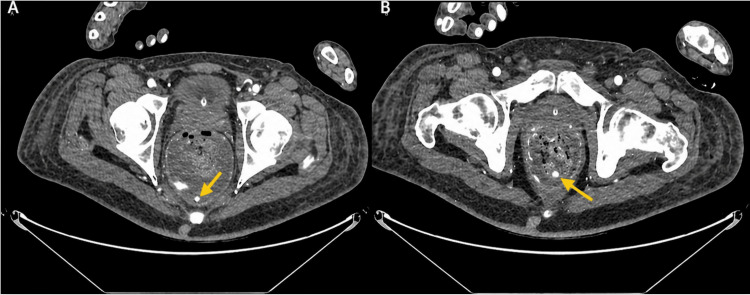
CT Angiography of the Pelvis Demonstrating Rectal Intraluminal Hyperdensity Concerning for Active Bleeding Axial contrast-enhanced CT images of the pelvis (A, B) demonstrate a distended rectum containing heterogeneous intraluminal material. A focal hyperdense intraluminal focus (arrows) is identified, concerning for contrast extravasation in the setting of acute hematochezia.

In the setting of ongoing hemorrhage and declining mental status, he was re-intubated for airway protection and transferred back to the ICU. His course was complicated by hemorrhagic shock requiring vasopressor support and activation of the massive transfusion protocol. 

Flexible sigmoidoscopy demonstrated ulcerated mucosa with stigmata of recent bleeding in the rectum and rectosigmoid colon (Figure 4), including a 3 cm ulcer located 20 cm from the anal verge and a 1 cm ulcer located 10 cm from the anal verge. Neither ulcer was actively bleeding at the time of the procedure; therefore, no endoscopic hemostatic intervention was performed. Lavage was carried out to remove residual stool and blood products and to improve visualization.

**Figure 4:**
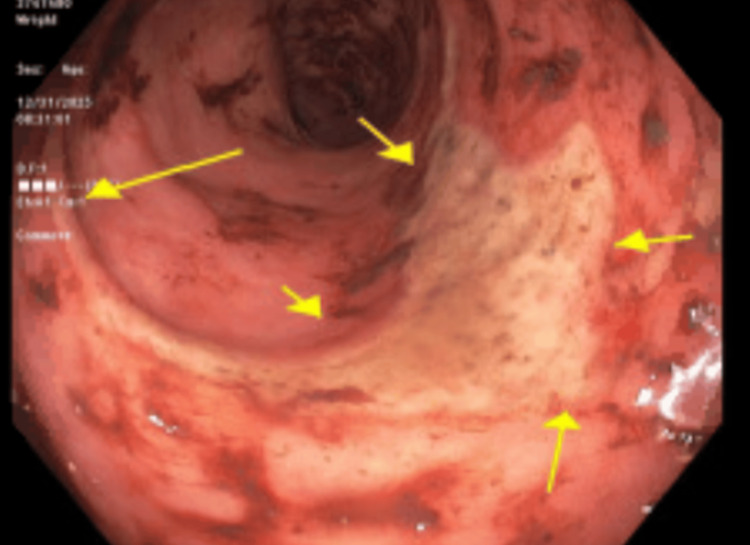
Flexible Sigmoidoscopy Demonstrating Rectal Ulceration Consistent with Acute Hemorrhagic Rectal Ulcer Endoscopic view of the rectum demonstrating multiple areas of ulcerated mucosa with surrounding erythema and stigmata of recent bleeding (arrows). The lesions are characterized by irregular, shallow ulcerations with overlying exudate, consistent with acute hemorrhagic rectal ulcer.

Following intervention, the patient achieved hemodynamic stabilization. ICU management included mechanical ventilation with serial ABG monitoring until mentation improved and the patient was successfully extubated. Temporary vasopressor support was required for one day to maintain a MAP >65 mm Hg, after which vasopressors were discontinued as blood pressure stabilized. Hemorrhagic management included close serial hemoglobin monitoring, IV fluid resuscitation, and transfusion support with packed red blood cells, platelets, and fresh frozen plasma as needed. All anticoagulation and antiplatelet therapies were held. Interventional Radiology and Colorectal Surgery were actively involved in management and recommended conservative treatment. IR angiography showed no active extravasation; therefore, no embolization was performed, and no surgical intervention was indicated. The patient was subsequently transferred out of the ICU after complete resolution of hematochezia for several days, stabilization of hemoglobin levels, and significant improvement in mentation, after which he returned to the medical floor. He continued to improve clinically and was ultimately discharged to a subacute rehabilitation facility.

## Discussion

Critically ill patients recovering from septic shock remain at risk for delayed ischemic complications, particularly with vasopressor exposure. New-onset gastrointestinal bleeding in this setting should prompt consideration of less common ischemic etiologies, including AHRU.

In the ICU, gastrointestinal bleeding is most often due to stress-related mucosal disease, particularly peptic ulcer disease. Accordingly, stress ulcer prophylaxis with histamine-2 receptor antagonists or proton pump inhibitors (e.g., famotidine, pantoprazole) is routinely used to reduce upper gastrointestinal bleeding [3]. Despite this, overt bleeding still occurs in approximately 2-4% of ICU patients [8].

Lower gastrointestinal bleeding is less common, and rectal ulcers are rare due to the rectum’s dual blood supply. AHRU, however, is a clinically significant entity; in one series of ICU patients with lower gastrointestinal bleeding, only 16.4% were attributed to AHRU [9].

We report a delayed presentation of AHRU-related bleeding in a patient without prior gastrointestinal disease following septic shock and prolonged vasopressor therapy. The absence of upper gastrointestinal bleeding, likely due to appropriate prophylaxis, highlights that current strategies may not prevent distal ischemic injury.

Splanchnic hypoperfusion is central to this process [4]. Hypovolemia, reduced cardiac output, and catecholamine-mediated vasoconstriction impair mucosal perfusion, disrupt protective barriers, and promote ischemia [10,11]. The rectosigmoid junction (Sudeck’s point), a watershed zone between the inferior mesenteric and superior rectal arteries, is particularly vulnerable during low-flow states [6,12]. Although colonic ischemia in sepsis more commonly affects other regions, rectal involvement at this site is recognized [6].

This case supports a “two-hit” model: initial hypoperfusion from septic shock primes the mucosa, while ongoing vasopressor use exacerbates regional ischemia, leading to ulcer formation. These sequential insults drive inflammation, endothelial dysfunction, and oxidative stress, increasing susceptibility to delayed injury [13].

This framework also underscores the limitations of current prophylaxis, which primarily targets upper gastrointestinal bleeding. AHRU likely reflects a multifactorial process involving hypoperfusion [5], systemic illness, and local factors such as immobility, constipation, and rectal devices. Opioid use further contributes to reducing bowel motility.

This case highlights the need to expand ICU gastrointestinal prevention strategies beyond peptic ulcer disease. Attention to bowel regimens, frequent patient repositioning, maintenance of adequate splanchnic perfusion, and careful use of rectal devices may help reduce risk. Frequent patient repositioning, typically performed every two hours in bedridden ICU patients, may help reduce prolonged pressure over dependent pelvic and rectal tissues, improve local perfusion, and decrease complications associated with prolonged immobility and tissue ischemia. Rectal devices, such as fecal management systems or rectal tubes, are commonly used in ICU patients for stool diversion, management of fecal incontinence, and prevention of skin breakdown; however, prolonged use may contribute to rectal mucosal injury, pressure necrosis, ulcer formation, ischemia, and lower gastrointestinal bleeding. Clinicians should maintain a high index of suspicion for ischemic causes of lower gastrointestinal bleeding in patients recovering from septic shock, even after apparent stabilization.

## Conclusions

This case illustrates AHRU as a rare but significant and serious complication causing delayed lower gastrointestinal bleeding in patients recovering from septic shock. Despite appropriate stress ulcer prophylaxis, ischemic injury may occur, particularly with prolonged vasopressor use. Clinicians should consider ischemic etiologies in new-onset bleeding in this population and recognize the need for broader preventive strategies beyond upper gastrointestinal protection. 
